# Recommendations for Neuromodulation in Diabetic Neuropathic Pain

**DOI:** 10.3389/fpain.2021.726308

**Published:** 2021-09-07

**Authors:** Zachary T. Olmsted, Amir Hadanny, Anthony M. Marchese, Marisa DiMarzio, Olga Khazen, Charles Argoff, Vishad Sukul, Julie G. Pilitsis

**Affiliations:** ^1^Department of Neuroscience and Experimental Therapeutics, Albany Medical College, Albany, NY, United States; ^2^Department of Neurosurgery, Albany Medical College, Albany, NY, United States; ^3^Department of Neurology, Albany Medical College, Albany, NY, United States

**Keywords:** neuropathic pain, painful diabetic neuropathy, diabetes mellitus, neuromodulation, pharmacotherapy, pain management, pain referral

## Abstract

Over 50% of the 34 million people who suffer from diabetes mellitus (DM) are affected by diabetic neuropathy. Painful diabetic neuropathy (PDN) impacts 40–50% of that group (8.5 million patients) and is associated with a significant source of disability and economic burden. Though new neuromodulation options have been successful in recent clinical trials (NCT03228420), still there are many barriers that restrict patients from access to these therapies. We seek to examine our tertiary care center (Albany Medical Center, NY, USA) experience with PDN management by leveraging our clinical database to assess patient referral patterns and utilization of neuromodulation. We identified all patients with a diagnosis of diabetes type 1 (CODE: E10.xx) or diabetes type 2 (CODE: E11.xx) AND neuralgia/neuropathic pain (CODE: M79.2) or neuropathy (CODE: G90.09) or chronic pain (CODE: G89.4) or limb pain (CODE: M79.6) OR diabetic neuropathy (CODE: E11.4) who saw endocrinology, neurology, and/or neurosurgery from January 1, 2019, to December 31, 2019. We then determined which patients had received pain medications and/or neuromodulation to divide the cohort into three groups: no treatment, conservative treatment, and neuromodulation treatment. The cohorts were compared with chi-square or one-way ANOVA with multiple comparisons to analyze the differences. A total of 2,635 PDN patients were identified, of which 700 received no treatment for PDN, 1,906 received medication(s), and 29 received neuromodulation (intrathecal therapy, spinal cord stimulation, or dorsal root ganglion stimulation). The patients who received pain medications for PDN visited neurology more often than the pain specialists. Of the patients that received neuromodulation, 24 had seen neurology, 6 neurology pain, and 3 anesthesia pain. They averaged 2.78 pain medications prior to implant. Approximately 41% of the patients in the conservative management group were prescribed three or more medications. Of the 1,935 treated patients, only 1.5% of the patients received neuromodulation. The patients on three or more pain medications without symptomatic relief may be potential candidates for neuromodulation. An opportunity, therefore, exists to educate providers on the benefits of neuromodulation procedures.

## Introduction

Diabetic neuropathies result from a multifaceted disease process and impact approximately half of diabetic patients worldwide (1). The pain associated with diabetic neuropathy is a significant source of patient disability and economic burden, with personal costs of up to US$7,066 more annually vs. patients without pain (2). In addition to early intervention and strict glycemic control, a variety of conservative/pharmacological and neuromodulatory treatment modalities have emerged for the management of painful diabetic neuropathy (PDN). The conservative treatment options include the common first-line neuropathic pain agents, such as pregabalin, gabapentin, and duloxetine (3, 4). Notably, the use of oral opioids is discouraged due to significant morbidity, mortality concerns, and lack of efficacy for neuropathic pain (5). Neuromodulatory interventions, such as intrathecal therapy (6), spinal cord stimulation (SCS) (7–9), and dorsal root ganglion stimulation (DRGS) (10), have been recently applied (11, 12).

Despite the availability of effective management strategies, the utilization of such treatments remains unclear. Given the complex nature of the diabetic neuropathic pain and the range of treatment options available, it is likely that an optimized and coordinated multi-disciplinary approach, such as neuromodulation, will be superior to management by any single discipline. Such a coordinated approach will benefit by focused evaluation of treatment utilization and efficacy to refine the future strategies and access to care.

This study aimed to explore the referral patterns of patients with PDN at a tertiary academic medical center with a multi-disciplinary pain practice to determine the utilization of neuromodulation as a treatment for neuropathic pain. We aimed to compare no treatment, conservative treatment, and neuromodulation treatment cohorts based on the diagnoses, referrals, therapeutic strategies, and/or demographic information.

## Materials and Methods

### Participants

**Figure 1:**
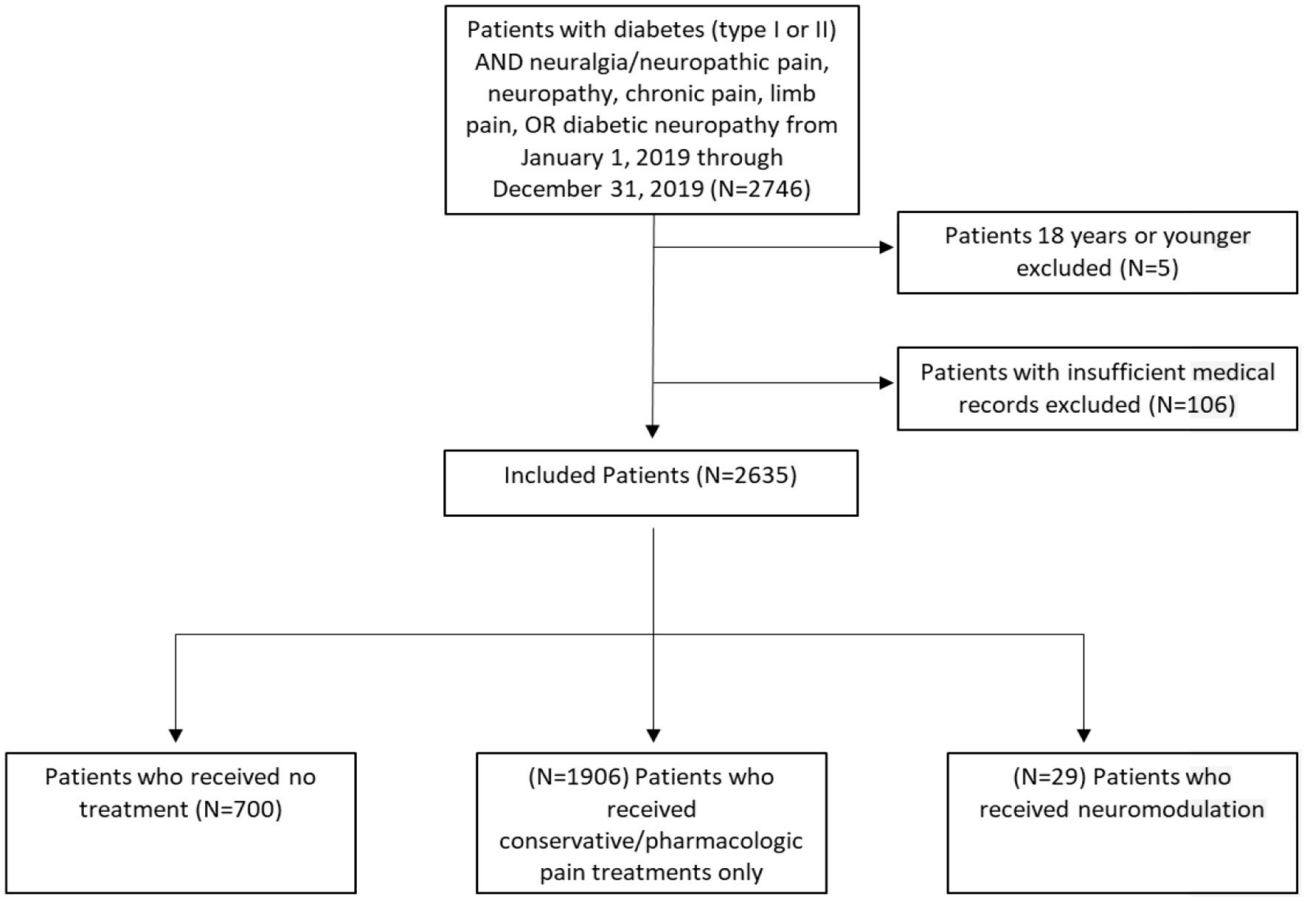
Flowchart of patient selection criteria.

To determine our experience with the referral patterns of patients with diabetic neuropathy, we partnered with our clinical bioinformatics team. Specifically, all patients who saw endocrinology, neurology, and/or neurosurgery at our tertiary care center between January 1, 2019, and January 2, 2020, were identified using the billing codes for a diagnosis of diabetes mellitus (DM) type 1 (CODE: E10.xx) or DM type 2 (CODE: E11.xx) AND neuralgia/neuropathic pain (CODE: M79.2) or neuropathy (CODE:G90.09) or chronic pain (CODE: G89.4) or limb pain (CODE: M79.6) OR diabetic neuropathy (CODE: E11.4). The notes of patients were manually examined to verify the diagnosis of painful neuropathy. We applied a custom bioinformatics platform to data from our clinical data warehouse to investigate the experience of our tertiary care center with PDN management based on referral patterns (Figure 1).

All patient charts were reviewed for usage of conservative treatment by gabapentin, pregabalin, duloxetine, venlafaxine, topiramate, carbamazepine, oxcarbazepine, amitriptyline, nortriptyline, lidocaine, capsaicin, and cannabis. Patients who underwent neuromodulation received either intrathecal therapy, SCS, or DRGS, which was determined using Current Procedural Terminology (CPT) codes (63655, 63685, 63650, 63650-51, 63688, 62350, 62362, 63664, and 62360). The study was approved by our institutional review board (IRB).

### Bioinformatics Pipeline Analysis

Following the retrieval of all patient identification numbers by CPT codes, data mining was performed to elicit the patient diagnoses, laboratories, medications, and provider meetings using SQL queries on the Albany Medical Center large data warehouse. Subsequently, a natural language processing algorithm was used to perform the text classification. We identified the patients with PDN diagnosis and confirmed by manual chart review. Finally, we retrieved all neuromodulation procedures performed and cross-referenced this list with these patients to enable separation into conservative treatment and neuromodulation cohorts.

### Study Design

In our total and neuromodulation cohorts identified in the billing code search, we gathered demographic information on age, sex, and DM type. The patient visits to providers from endocrinology, general neurology, pain neurology (anesthesia), and/or neurosurgery were documented. The number of past and current prescribed pain medications per patient was recorded.

### Statistical Analysis

Comparative differences in the utilization of various PDN treatment options were analyzed using correlation analyses, unpaired *t*-tests, and chi-squares. The demographic data of the three cohorts were compared using chi-square, unpaired *t*-test, or one-way ANOVA with Tukey's correction for multiple comparisons. Data were analyzed using GraphPad Prism 8 (GraphPad Software, San Diego, CA, USA). A *p* < 0.05 was considered to be statistically significant.

## Results

### Applying a Bioinformatics Pipeline to Elicit Patient Selection Criteria

**Figure 2:**
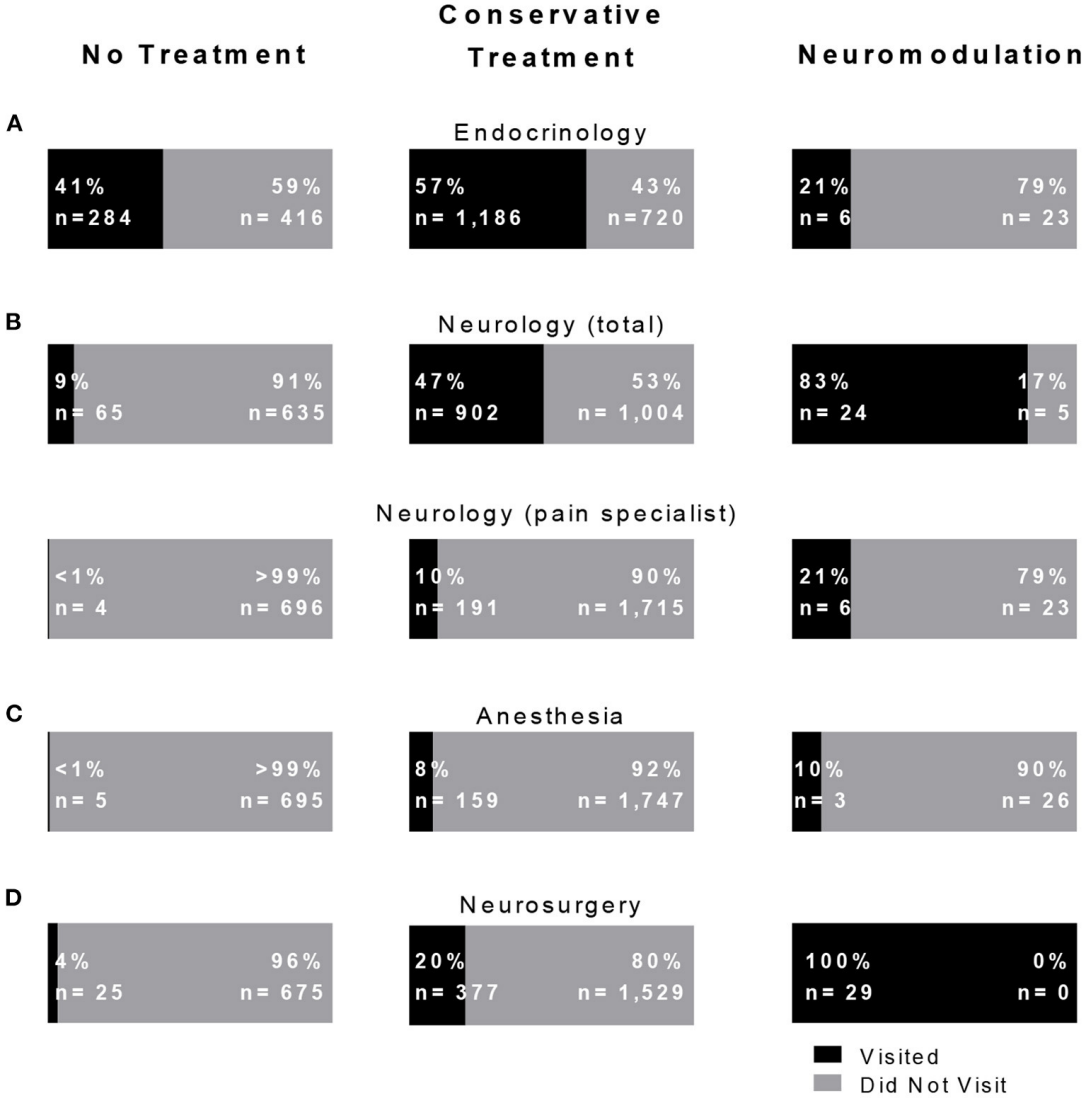
Non-conservatively and conservatively treated painful diabetic neuropathy (PDN) patients visit endocrinology but not neurosurgery. The percentage of PDN patient per treatment group who visited to **(A)** endocrinology, **(B)** neurology (total) and the subset of neurology that visited neurology pain specialists, **(C)** anesthesia, and **(D)** neurosurgery.

**Figure 3:**
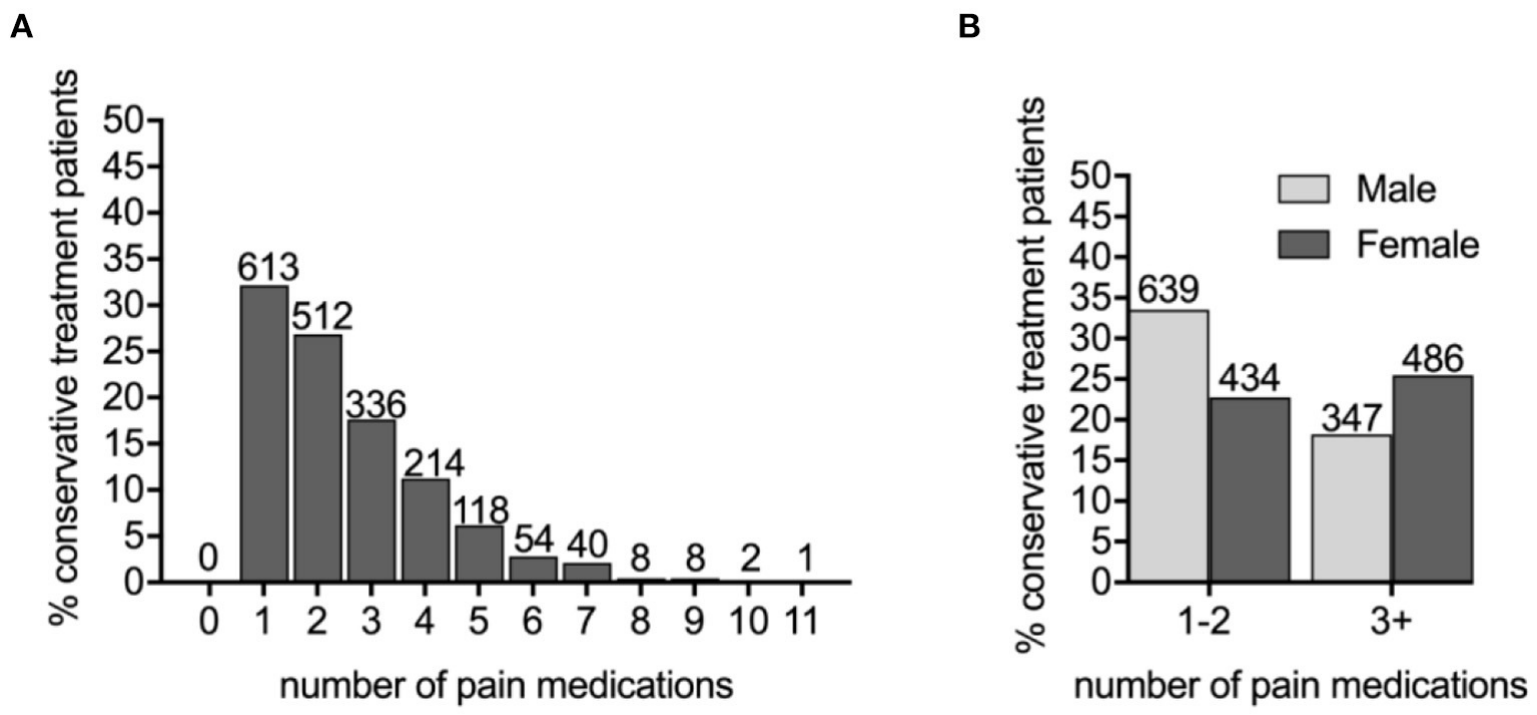
The PDN patients prescribed three or more pain medications are more likely to be female. **(A)** The number of different pain medications taken by conservatively treated PDN patients. **(B)** Male and female conservatively treated patients were prescribed one–two or three or more pain medications (X^2^ = 28.2479, *p* < 0.0001).

In 1 year, 2,746 patients with DM (type 1 or 2) were treated for neuralgia/neuropathic pain, neuropathy, chronic pain, limb pain, or diabetic neuropathy at our center. Patients aged 18 years old or younger (*N* = 5) and those with no data in their medical records were excluded (*N* = 106), leaving a total dataset of *N* = 2,635 patients for further analysis. The total included patient population was further separated into three cohorts: (1) no treatment (*N* = 700), (2) treatment with conservative/pharmacologic pain medications only (*N* = 1,906), and (3) treatment with neuromodulation (*N* = 29). The three cohorts were compared on the basis of sex, age, diabetes status (DM type 1 or 2) (Table 1), patient provider visits (Figure 2), and pain medication usage (Figure 3).

**Table 1:** Patient demographics among each cohort.

	**No treatment**	Conservative treatment^*^	**Neuromodulation^†^**
**Sex**			
Female	282/700 (40.29%)	920/1,906 (48.27%)	14/29 (48.28%)
Male	418/700 (59.71%)^***^	986/1,906 (51.73%)	15/29 (51.72%)
**Age (±SEM)**	65.11 ± 0.50^***^	63.08 ± 0.29	63.69 ± 2.05
**Diabetes**			
Type 1	49/700 (7.00%)	202/1,906 (10.60%)	1/29 (3.45%)
Type 2	651/700 (93.00%)	1,704/1,906 (89.40%)	28/29 (96.55%)

* *Conservative treatment: Gabapentin, pregabalin, duloxetine, venlafaxine, topiramate, carbamazepine, oxcarbazepine, amitriptyline, nortriptyline, lidocaine, capsaicin, and cannabis*.

† *Neuromodulation: Intrathecal therapy, spinal cord stimulation, and dorsal root ganglion stimulation*.

*** *p < 0.001*.

### Comparative Analysis of Cohort Demographics

Sex, age, and diabetes type (type 1 or type 2) among the three cohorts were compared (Table 1). We determined that the untreated PDN patients (65.11 ± 0.50 years) were more likely to be older than patients on conservation management (63.08 ± 0.29) and more likely to be male (59.71%) than the conservatively treated patients (51.73%). Specifically, age differed between the no treatment and conservative treatment groups with Tukey's multiple comparison test (mean difference, 95% *CI* of difference, adjusted *p*-value 2.061 [0.726–3.397], *p* < 0.001). Age was not statistically significant between untreated and neuromodulation cohorts. Sex and DM type differed using chi-square analysis between the no treatment and conservative treatment cohorts only (X^2^ = 13.13, *p* < 0.001; X^2^ = 7.62, *p* = 0.006, respectively). Patients prescribed three or more pain medications were more likely to be female (X^2^ = 28.25, *p* < 0.001) (Figure 3B) and were slightly younger (aged 61.51 ± 0.43 years for 3+ group compared with 64.17 ± 0.38 in the 0–2 medications group *p* < 0.001). For the no treatment group, 651/700 (93.00%) patients carried a diagnosis of type 2 DM vs. 1,704/1,906 (89.40%) patients on the conservative management.

### Comparative Analysis of PDN Patient Specialty Provider Visits

We applied our bioinformatics pipeline to delineate provider visits for the three treatment cohorts (Figure 2). Visits to the medical provider specialties of endocrinology, neurology (total), general neurology, pain neurology, anesthesia (pain), and neurosurgery (all providers) were analyzed. There were significant differences among each treatment cohort in visits to general (total) neurology (no treatment 9%, conservative treatment 47%, and neuromodulation 83%, X^2^ = 341.795, *p* < 0.001), neurology pain (no treatment < 1%, conservative treatment 10%, and neuromodulation 21%, X^2^ = 71.98, *p* < 0.001), and to anesthesia pain (no treatment <1%, conservative treatment 8%, and neuromodulation 10%, X^2^ = 83.31, *p* < 0.001). Compared with no treatment patients, the conservative treatment patients more frequently saw endocrinology (X^2^ = 97.62, *p* < 0.001), neurology (X^2^ = 317.42, *p* < 0.001), anesthesia pain (X^2^ = 50.51, *p* < 0.001), and neurosurgery (X^2^ = 103.09, *p* < 0.001). Of the patients that received neuromodulation, 24 had seen general neurology, 6 pain neurology, and 3 anesthesia pain specialists.

### Pain Medication Usage as a Potential Neuromodulation Candidate Identifier

**Figure 4:**
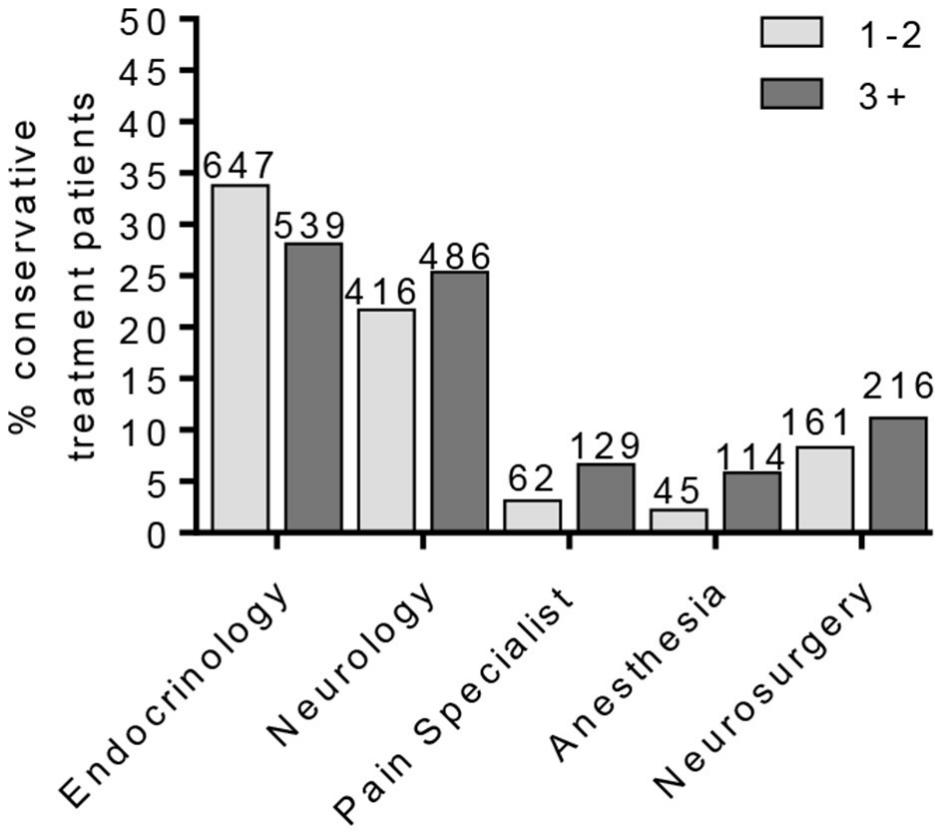
Percentage of conservative treatment patients seen by providers. The percentage of PDN patients in the conservative treatment group according to patient provider visits. This cohort was further separated into patients on one–two or three or more pain medications.

**Figure 5:**
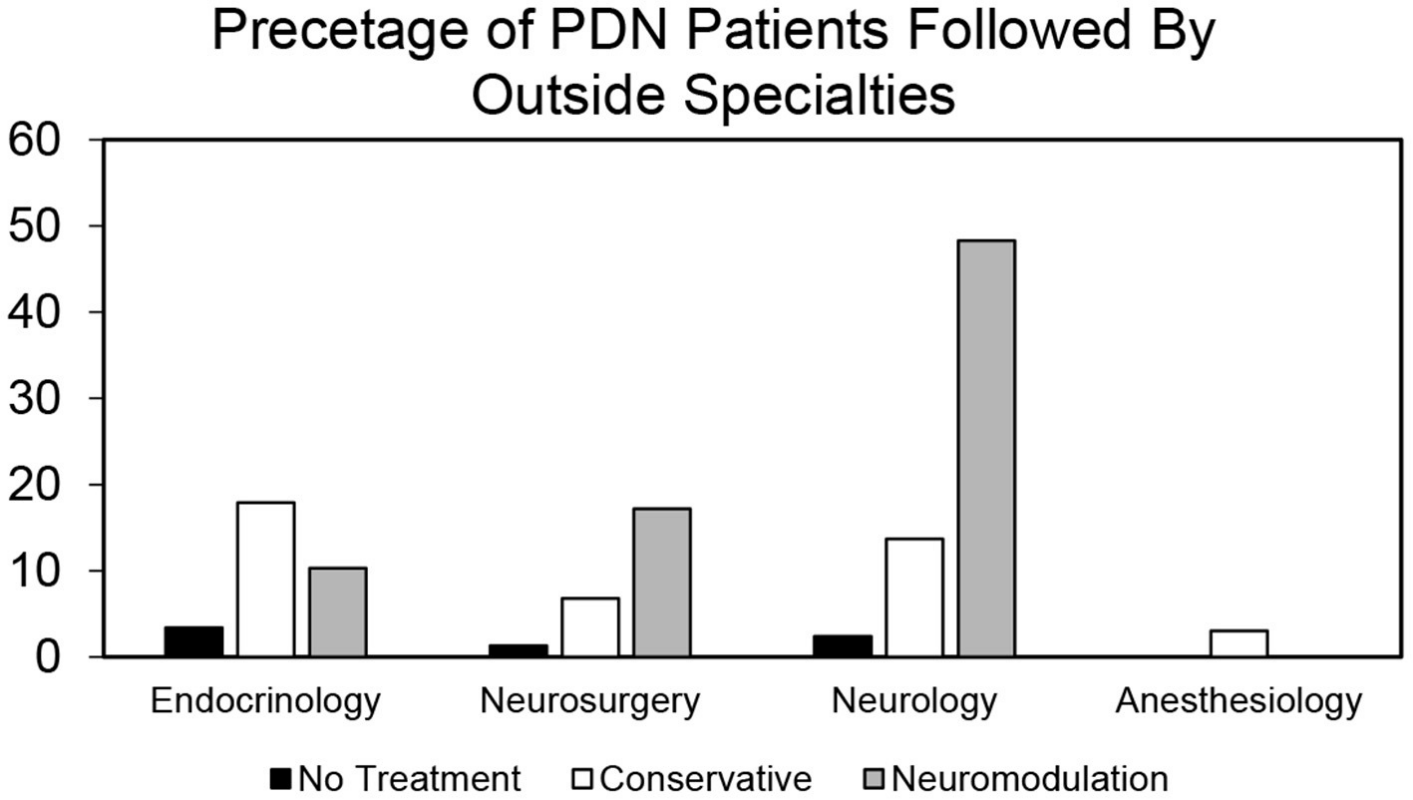
Proportion of patients by provider type who are followed by other specialties in no treatment, conservative treatment, and neuromodulation cohorts. Histogram of percentage of patients from all the three cohorts that are followed by other specialties in addition to their primary provider as determined by our bioinformatics pipeline.

To identify a population of patients on the conservative pain management that may be candidates for neuromodulation therapy, we divided these patients into two groups: patients that were prescribed one–two medications and patients that were prescribed three or more medications. We chose three or more medications since this is typically the number of medications that the patients must fail to respond to in a neuromodulation clinical trial design. Patients who have tried multiple medications may be candidates for neuromodulation (Figure 3). In the conservative management cohort, 59.03% of the patients were prescribed 1–2 medications, while 40.97% were on 3 or more pain medications. Of patients referred for neuromodulation, 14/29 were on 3 or more medications. The cohort as a whole averaged 2.78 medications (Figure 4). The percentage of patients that were followed by other specialties in addition to their primary provider is provided in Figure 5. Neuromodulation patients seen by endocrinology, neurosurgery, and neurology were often followed by additional specialties (10.3, 17.2, and 48.3%, respectively). The conservatively managed patients had lower percentages (17.9, 6.8, and 13.7%, respectively). We observed that very few PDN patients with no treatment were followed by multiple specialties. In addition, anesthesiology saw few PDN patients.

## Discussion

Diabetes mellitus affects a substantial proportion of the global population (34 million) (1). Of these patients, approximately 50% are affected by diabetic neuropathic sequalae that can progress to chronic PDN (~8.5 million), significantly reducing quality of life and constituting a substantial global health cost (2). While diabetic patients are often clinically managed by endocrinology, the widespread prevalence of chronic neurological symptoms necessitates a multi-disciplinary approach to long-term pain management. Traditionally, the neuropathic symptoms are managed with non-opioid pain medications (3, 4). However, with the advent of neuromodulation therapy either by intrathecal drug delivery (6) or by electrical stimulation of neuroanatomical therapeutic sites (7–11), the landscape of therapeutic approaches available is expanding. This is particularly true for PDN patients with pain symptoms that are chronic and refractory to conservative pharmacological management. In a recent systematic review and meta-analysis investigating the use of invasive electrical neuromodulation for PDN (11), Raghu et al. provided comprehensive evidence that neuromodulation, such as SCS and DRGS, is an efficacious, safe, and long-lasting option. Recently, Petersen et al. reported the results of a randomized clinical trial investigating the effect of high-frequency SCS on PDN pain management in 216 patients. This trial produced promising outcomes for the efficacy and safety of SCS, wherein substantial pain relief and improved quality of life measures resulted and were sustained over 6 months (13). To fully elucidate the utilization of this intervention, we examined the experience of our tertiary care center with PDN management by applying a bioinformatics pipeline to our clinical data warehouse and assessed patient referral patterns and treatment type.

We identified a total of 2,635 PDN patients. Of these cases, 700 received no treatment for PDN, 1,906 received medication, and 29 received neuromodulation. Effectively, only 1.5% of the treated patients received neuromodulation over the 1-year period that we analyzed. A patient cohort referred to neurosurgery was more similar in age and sex to the conservative treatment cohort than the no treatment cohort.

Using the criterion of patients that are on three or more pain medications without symptomatic relief, we stratified a group of conservatively treated patients as candidates for neuromodulation therapy. This benchmark is the typical for medication trial and failure prior to neuromodulation in a clinical trial design. Indeed, it is not atypical for chronic pain patients to be prescribed even 6–11 pain medications over time as reflected in our data. We identified nearly 41% of the conservatively treated patients having prescriptions for more than three pain medications. These patients were more likely to be female (14, 15) and were also younger than those prescribed two or less medications. All treatment cohorts were more likely to have DM type 2 as compared to DM type 1. This would be expected given the incidence of DM type 2 (16) and the time required for the onset of neurological symptoms (17).

Neuromodulation therapies are ever-expanding with increased efficacy and indications for long-term management of chronic pain. These advances are outpacing those new non-opioid pharmaceuticals for pain management and have lower potential for abuse. This study aimed to understand the number of potential candidates for neuromodulation and the providers they visited. Endocrinology appears to be referring patients adequately for PDN management based on their number of visits and the number of patients that go for neurology. Further, 24/29 patients came from neurology providers, in contrast to 5 that were seen by a pain neurology provider. An opportunity to partner with general neurology for care of these patients in neuromodulation exists. A potential exists for more than 40% additional patients to be referred. It should be noted, however, that these medications may have been prescribed for symptoms related to pain disorders other than PDN, such as headache or back/neck related symptoms. As we have no way of parsing that information, consideration of prescribed pain medications may aid in the identification of potential neuromodulation referrals.

Although our study discusses important findings on the medical management of PDN and the utilization of neuromodulation for PDN patients, there are several limitations. While a large sample size is used (*N* = 2,635 total), the study is observational and future studies, therefore, should plan to prospectively evaluate the efficacy of neuromodulation for PDN. The study investigates a 1-year time period. The number of subjects in the neuromodulation cohort (*N* = 29) was small with respect to the no treatment (*N* = 700) and conservative treatment (*N* = 1,906) cohorts, yielding lower statistical power. However, this low number also reflects the underutilization of neuromodulation therapy for PDN, and we establish a significant proportion of patients undergoing the conservative treatment as neuromodulation candidates. Many older patients with multiple comorbidities may have treatment failure but cannot take multiple medications due to poor tolerance to side effects or drug interactions, which may have impacted our results in these age groups. It will be informative in future studies that apply a modified bioinformatics data handling and analysis pipeline to document the temporal sequence of patient progression from one provider to another during management to determine the critical points where multi-disciplinary intervention can be optimized.

## Conclusions

We aimed to explore the referral patterns of PDN patients at our tertiary academic medical center that contains a multi-disciplinary pain practice to determine the utilization of neuromodulation as a treatment for neuropathic pain. The majority of patients with PDN were referred from endocrinology to general neurology, and 40% of those patients were on multiple medications and may be candidates of neuromodulation therapy. These results, therefore, warrant education of providers regarding the potential benefits of neuromodulation procedures.

## Data Availability Statement

The datasets presented in this article are not readily available because the dataset generated and analyzed in the current study is comprised of private patient information, gathered from patient charts, and therefore is not publicly available. The anonymized dataset can be made available from the corresponding author upon reasonable request. Requests to access the datasets should be directed to jpilitsis@yahoo.com.

## Ethics Statement

The studies involving human participants were reviewed and approved by Institutional Review Board at Albany Medical Center. Written informed consent for participation was not required for this study in accordance with the national legislation and the institutional requirements.

## Author Contributions

JP conceived and coordinated the study, provided patient care, directed chart review and analysis, and assisted in writing the manuscript. ZO, AH, AM, OK, and MD assisted in writing the manuscript. AM, ZO, and AH composed figures and tables. CA and VS provided patient care and patient data. AM, AH, MD, and OK performed chart review and statistical analysis. AH developed the bioinformatics pipeline for analysis of chart data. All authors contributed to manuscript editing and approved the final submission.

## Conflict of Interest

JP is a consultant for Boston Scientific, Nevro, Medtronic, Saluda, and Abbott and receives grant support from Medtronic, Boston Scientific, Abbott, Nevro, NIH 2R01CA166379-06, and NIH U44NS115111. She is the medical advisor for Aim Medical Robotics and Karuna and has stock equity. VS is a consultant for Boston Scientific and Medtronic. CA is a consultant for Teva, Lilly, Allergan, Amgen, Novartis, and Flowonix. He provides research support to Teva and Allergan. He has stock in Pfizer. He is part of the Speaker's Bureau for Theranica, Tercera, Teva, Lilly, Allergan, Amgen, Novartis, and Flowonix. AH has stock equity in EEG Sense and Aviv Scientific. The remaining authors declare that the research was conducted in the absence of any commercial or financial relationships that could be construed as a potential conflict of interest.

## Publisher's Note

All claims expressed in this article are solely those of the authors and do not necessarily represent those of their affiliated organizations, or those of the publisher, the editors and the reviewers. Any product that may be evaluated in this article, or claim that may be made by its manufacturer, is not guaranteed or endorsed by the publisher.
